# Crystal Structure of the *Escherichia coli* Fic Toxin-Like Protein in Complex with Its Cognate Antitoxin

**DOI:** 10.1371/journal.pone.0163654

**Published:** 2016-09-22

**Authors:** Frédéric V. Stanger, Alexander Harms, Christoph Dehio, Tilman Schirmer

**Affiliations:** 1 Focal Area Structural Biology and Biophysics, Biozentrum, University of Basel, Basel, Switzerland; 2 Focal Area Infection Biology, Biozentrum, University of Basel, Basel, Switzerland; University of Manchester, UNITED KINGDOM

## Abstract

FIC domain proteins mediate post-translational modifications of target proteins, which typically results in their inactivation. Depending on the conservation of crucial active site residues, the FIC fold serves as structural scaffold for various enzymatic activities, mostly target adenylylation. The founding member of the vast Fic protein family, EcFicT, was identified in *Escherichia coli* some time ago. The G55R point mutant of EcFicT displays the “filamentation induced by cAMP” (Fic) phenotype at high 3',5'-cyclic adenosine monophosphate (cAMP) concentrations and elevated temperature, but the underlying molecular mechanism and any putative biochemical activity of EcFicT have remained unknown. EcFicT belongs to class I Fic toxin proteins that are encoded together with a small inhibitory protein (antitoxin), named EcFicA in *E*. *coli*. Here, we report the crystal structures of two mutant EcFicT/EcFicA complexes (EcFicT_G55R_A and EcFicTA_E28G_) both showing close resemblance with the structure of the AMP-transferase VbhT from *Bartonella schoenbuchensis* in complex with its cognate antitoxin VbhA. However, crucial differences in the active site of EcFicT compared to VbhT and other AMP-transferases rationalize the lack of evidence for adenylylation activity. Comprehensive bioinformatic analysis suggests that EcFicT has evolved from canonical AMP-transferases and has acquired a conserved binding site for a yet to be discovered novel substrate. The G55R mutation has no effect on structure or thermal stability of EcFicT, such that the molecular basis for its associated Fic phenotype remains elusive. We anticipate that this structure will inspire further bioinformatic and experimental analyses in order to characterize the enzymatic activity of EcFicT and help revealing its physiological role.

## Introduction

Filamentation induced by cAMP (Fic) was discovered in the early 1980s by Utsumi *et al*. [1] as a phenotype caused by the temperature sensitive allele *fic-1* of the *fic* gene of *Escherichia coli*. The G55R mutation encoded by the *fic-1* allele caused cell filamentation at non-permissive temperature (43°C) in the presence of high concentration of cAMP (1.5 mM) in the culture medium [2–5]. In their reports, they showed that knock-out strains of the *fic* gene were viable as wild-type. In contrast, bacteria expressing the *fic-1* allele (G55R) displayed a filamentation phenotype, suggesting a gain of function mutation [3,4]. A complementation of the *Δfic* knock-out mutant with the *fic-1* allele also resulted in bacterial filamentation. Yet, the biochemical function of the *E*. *coli* Fic protein remained unclear.

A few years ago, a biochemical activity likely shared by the majority of Fic proteins has been identified by *in vitro* and *in vivo* analyses combined with mass spectrometry [6–9]. Representative members of FIC domain proteins containing the canonical HxFx[D/E]GNGR_1_xxR_2_ motif were found to catalyze adenylylation (also known as AMPylation), the transfer of an AMP moiety from the ATP substrate onto a hydroxyl side-chain of a target protein. Crystal structures showed that the ATP substrate interacts directly with arginines R_1_ and R_2_ and with the central asparagine [9]. In addition, the substrate is indirectly bound to the aspartate or glutamate residue of the Fic active site motif *via* a magnesium ion. The histidine is thought to constitute the general base for deprotonation of the target hydroxyl side chain to allow in-line nucleophilic attack onto the α-phosphorous of ATP. However, Fic proteins are versatile enzymes in that they can also catalyze other post-translational modifications depending on their active site signature motif and the nature of the substrate binding pocket. Recent studies reported phosphocholination and phosphorylation (kinase) activity for AnkX [10] and Doc [11, 12], respectively.

Catalytic activity is usually inhibited by a conserved inhibitory α-helix (α_inh_) that partly obstructs the ATP binding site. The α_inh_ can either be part of the same polypeptide chain (located relative to the Fic domain N-terminal in class II Fic proteins and C-terminal in class III Fic proteins, respectively) or provided by a separate small protein known as the antitoxin (class I Fic proteins) [9]. The α_inh_ helix contains a [S/T]xxxE[G/N] sequence motif with the invariant glutamate forming a salt-bridge with R_2_ of the FIC motif [9].

Structure/function studies on class I Fic proteins (designated as FicT toxins) and their cognate antitoxins (designated as FicA) have focused so far on the toxin-antitoxin (TA) module VbhTA of *Bartonella schoenbuchensis* [9,13–15]. VbhT was shown to interfere with bacterial growth by adenylylation and concomitant inactivation of DNA gyrase and topoisomerase IV [15]. The VbhA antitoxin inhibits the catalytic activity of VbhT by direct interaction, and *vhbA-vbhT* is encoded in an operon like other *ficA*-*ficT* gene pairs. VbhTA and other FicTA systems thus satisfy the key definition of a type II TA [15].

The *fic* gene of *E*. *coli* (renamed as *ecficT*) is preceded by the small upstream gene *yhfG* (renamed as *ecficA*) that encodes a FicA antitoxin homolog and thus shares typical characteristics of FicTA modules. The *bona fide* antitoxin EcFicA contains a canonical [S/T]xxxE[G/N] inhibition motif and has been shown to interact with EcFicT [15,16]. Even upon mutation of the inhibition motif, we were unable to demonstrate any auto- or target-adenylylation activity catalyzed by EcFicT [15]. Similarly, any cellular target(s) and the mechanisms underlying “filamentation induced by cAMP” have remained unknown.

Here, we describe the crystal structure of EcFicT in complex with its cognate antitoxin EcFicA. Phylogenetic analysis reveals that enterobacterial FicT toxins constitute a distinct subgroup that originated by vertical transmission and may have evolved a new molecular and biological function. The crystal structure of the EcFicT/EcFicA complex reveals an extended ligand-binding pocket that is strictly conserved within this EcFicT-like subfamily. The precise definition of this newly observed substrate-binding pocket will serve as a basis for *in silico* docking experiments that will guide potential ligand identification and further bioinformatical and evolutionary analyses of the Fic toxin-antitoxin family.

## Materials and Methods

### Phylogenetic analysis

The *E*. *coli* K12 MG1655 FicT protein (EcFicT; Uniprot: P20605) was used as reference sequence for comparison with similar protein sequences (>50% identity) from the UniProt Reference Clusters (UniRef) (702 sequences). For the phylogenies shown in the Supporting Information, the protein list was shortened to 107 sequences by removing redundant entries. For the phylogenies shown in the main figures, the protein list was further reduced to 47 sequences by applying a cut-off of 90% identity. Sequence alignments were created using ClustalW (implemented in Geneious v7.1.7; Biomatters) and manually curated. Subsequently, gapped positions were removed and a maximum likelihood phylogeny was constructed using PhyML implemented in Geneious (LG substitution model as suggested by ProtTest3 [17], 100 bootstraps), including two distant class I Fic proteins, YeFicT of *Yersinia enterocolitica* str. 8081 and VbhT of *B*. *schoenbuchensis*. The phylogeny was rooted using VbhT as outgroup. Phylogeny illustrations were processed using FigTree v1.4.2 (http://tree.bio.ed.ac.uk/software/figtree). The sequence dataset described above was submitted to the SPEER server using the SCI-PHY algorithm for automated subgrouping and a relative entropy value of 1.0 [18].

WebLogos for subgroups 1 and 3 were generated using the WEBLOGO server [19]. Additionally, the overall conservation was monitored.

Analysis of the genetic loci of EcFicT homologs was performed using the www.MicrobesOnline.org web tool [20] with a limit at 50 homologs and a clustering at 95% identity. Only one representative locus is shown per species.

### Cloning, expression and purification

Plasmids pPE0038 and pFVS0130 containing the *E*. *coli ecficT/ecficA* and *ecficT/ecficA*_*E28G*_ genes, respectively, were cloned in the pRSFDuet-1 vector (Novagen) as described previously [15]. Additionally, a single base-pair mutation was introduced in plasmids pPE0038 and pFVS0130, resulting in plasmids pFVS0129 and pFVS0315 containing the *ecficT*_*G55R*_/*ecficA* genes and *ecficT*_*G55R*_/*ecficA*_*E28G*_, respectively. EcFicT is fused to an N-terminal His_6_-tag and EcFicA to an N-terminal HA-tag. Plasmids pPE0038, pFVS0129, pFVS0130 and pFVS0315 were transformed into *E*. *coli* BL21 (DE3). EcFicT/EcFicA (Uniprot: P20605/P0ADX5) protein complexes were expressed and purified as described previously for VbhTA proteins [9] using the same buffer systems. EcFicT_G55R_A and EcFicTA_E28G_ were concentrated to 25 and 28 mg/mL, respectively.

### Differential scanning fluorimetry analysis

Temperature-induced unfolding was monitored by differential scanning fluorimetry experiments [21] performed on a Qiagen Rotor-gene Q real-time thermocycler as described previously [22].

### Crystallization

Crystals were obtained at 20°C using the sitting-drop vapour diffusion method after mixing 0.2 μL protein solution with 0.2 μL reservoir solution equilibrating against a reservoir of 80 μL. EcFicT_G55R_A crystallized after 5 days in 0.2 M MgCl_2_, 0.1 M Tris pH 8.0 and 20% (w/v) PEG 6000 and EcFicTA_E28G_ crystallized after 8 days in 19% PEG 1500, 0.1 M MMT (malic acid, MES, Tris) buffer pH 6.0.

### Data collection and processing

For data collection, crystals were cryo-protected by transfer into a reservoir solution supplemented with 20% glycerol and subsequently vitrified in liquid nitrogen. X-ray data were collected at the Swiss Light Source (Villigen, Switzerland) on beamline X06SA (PXI) and X06DA (PXIII) at 100 K and a wavelength of 1.000 Å. Diffraction data were indexed and integrated using XDS [23] and subsequently merged and scaled using XSCALE [23] or AIMLESS [24]. EcFicT_G55R_A and EcFicTA_E28G_ diffracted to 2.0 Å and 2.4 Å, respectively. Data collection statistics are summarized in Table 1.

**Table 1:** Data collection statistics.

	EcFicT_G55R_A	EcFicTA_E28G_
PDB code	5JFF	5JFZ
X-ray source	SLS X06DA (PXIII)	SLS X06SA (PXI)
X-ray detector	Pilatus 2M	Pilatus 6M
Wavelength (Å)	0.9999	1.0000
Space group	P 6_5_	C 2
Cell dimensions		
*a*, *b*, *c* (Å)	104.7, 104.7, 110.5	154.78, 64.40, 87.88
*β* (°)		113.8
Matthews coefficient (Å^3^/Da)	3.1	2.51
Solvent content (%)	60.5	50.6
EcFicTA complexes per a.u.	2	3
Resolution limits (Å)	91–2.00 (2.07–2.00)	80–2.40 (2.49–2.40)
R_merge_ † (%)	9.7 (86.5)	16.2 (66.5)
R_meas_ ‡ (%)	10.3 (97.3)	17.7 (73.8)
CC 1/2	99.9 (83.2)	99.4 (75.7)
⟨I / σ(I)⟩	22.7 (2.9)	8.7 (2.0)
Total reflections	472’112 (43’939)	192’193 (16’376)
Unique reflections	46’196 (4’553)	30’850 (2’657)
Multiplicity	10.2 (9.7)	6.2 (6.2)
Completeness (%)	99.7 (98.7)	98.6 (85.8)
Mosaicity	0.14	0.25

Numbers in parentheses belong to the outer shell.

† R_merge_ = ∑_hkl_∑_i_ |I_i_(hkl)—⟨I(hkl)⟩| / ∑_hkl_∑_i_ I_i_(hkl), where I_i_(hkl) is the observed intensity for a reflection and ⟨I(hkl)⟩ is the average intensity obtained from multiple observations of symmetry-related reflections.

‡ R_meas_ = ∑_hkl_ [N/(N-1)]^1/2^ ∑_i_ |I_i_(hkl)—⟨I(hkl)⟩| / ∑_hkl_∑_i_ I_i_(hkl), where I_i_(hkl) is the observed intensity for a reflection, ⟨I(hkl)⟩ is the average intensity obtained from multiple observations of symmetry-related reflections and N is the number of observations of intensity I(hkl).

### Structure determination

The structures were solved by molecular replacement using PHASER [25]. The initial model of the antitoxin was built using AUTOBUILD of the PHENIX package [26]. The complex models were improved by iterative rounds of interactive model building using COOT [27] and refinement using REFMAC5 [28] or PHENIX.REFINE [29]. Final refinement statistics are given in Table 2.

**Table 2:** Refinement statistics.

	EcFicT_G55R_A	EcFicTA_E28G_
PDB code	5JFF	5JFZ
Resolution limits (Å)	90.66–2.00 (2.07–2.00)	80.41–2.40 (2.49–2.40)
R_work_ * (%)	17.7 (23.4)	21.7 (27.7)
R_free_ ** (%)	20.4 (28.7)	26.8 (35.1)
Number of non-hydrogen atoms	4’294	5’560
*proteins*	3’887	5’413
*ligands*	6	0
*solvent*	401	147
Completeness of model		
EcFicT (residues)	10–15, 17–198	10–81, 88–195
EcFicA (residues)	3–53	5–53
Protein residues	478	686
R.m.s.d from ideal		
*Bond lengths (Å)*	0.019	0.013
*Bond angles (°)*	1.73	1.53
Ramachandran favored *** (%)	99	99
Ramachandran outliers *** (%)	0	0
Clashscore ***	3.12	2.92
Average B values (Å^2^)	30.20	33.70
*macromolecules*	29.60	33.80
*ligands*	40.50	n.a.
*solvent*	35.70	29.90

Numbers in parentheses refer to the outer shell.

* *R*_*work*_ = ∑_hkl_|| F_obs_|—|F_calc_|| / ∑_hkl_|F_obs_|

** R_free_ is the R value calculated for 5% of the data set that was not included in the refinement.

*** Molprobity

### Accession numbers

Coordinates and structure factors have been deposited in the Protein Data Bank with accession numbers 5JFF and 5JFZ for EcFicT_G55R_A and EcFicTA_E28G_, respectively.

## Results

### EcFicT and EcFicA form a stable complex

**Fig 1:**
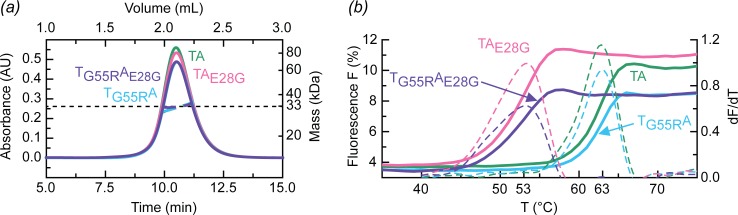
Analysis of the various EcFicTA complexes in solution. (*a*) SEC-MALLS analysis of EcFicTA (green), EcFicTA_E28G_ (pink), EcFicT_G55R_A (cyan) and EcFicT_G55R_A_E28G_ (purple) reveals in each case a 1:1 complex in solution at an eluting concentration of 15 μM. (*b*) Differential scanning fluorimetry (DSF) analysis of EcFicTA variants. Melting curves (continuous lines) and their first derivatives (broken lines) are shown. Complexes with the E28G mutation in the antitoxin show decreased stability compared to the wild-type complex, whereas the G55R mutation does not affect the thermal stability of the complex.

Co-expression of *ecficT* and *ecficA* in *E*. *coli* BL21 (DE3) yielded over 15 mg of soluble complex per liter of culture. Size exclusion chromatography coupled to multi-angle laser light scattering (SEC-MALLS) analyses revealed that all investigated constructs—wild-type EcFicT_wt_/EcFicA_wt_ complex (EcFicTA), single mutants EcFicT_G55R_/EcFicA_wt_ (EcFicT_G55R_A) and EcFicT/EcFicA_E28G_ (EcFicTA_E28G_), and double mutant EcFicT_G55R_/EcFicA_E28G_ (EcFicT_G55R_A_E28G)_—form a stable 1:1 complex, with a molecular mass between 32.1 kDa to 32.9 kDa, which closely corresponds to the theoretical mass of 33.4 kDa (Fig 1A).

Purified EcFicTA complexes were subjected to differential scanning fluorimetry (DSF) to determine their melting temperature (T_m_). Wild-type EcFicTA appears rather stable with a T_m_ of 63°C (green curve, Fig 1B). The G55R mutation that has been shown originally to cause *in vivo* the Fic phenotype [1] does not affect the melting temperature of the complex (EcFicT_G55R_A variant, blue curve, Fig 1B) despite additional intra-molecular interactions (see below). The melting curve appears monophasic. Thus, it is likely that the two proteins melt cooperatively, but we cannot exclude that both proteins have similar individual melting temperature. Strikingly, mutation of the putative inhibitory glutamate residue E28 (EcFicA_E28G_) decreases the melting temperature of the complex by 10°C compared to the wild-type complex (Fig 1B). The peaks of the first derivative of the melting curve of EcFicTA_E28G_ and EcFicT_G55R_A_E28G_ appear to broaden towards lower temperature, indicating that the E28G mutation may weaken the complex stability, resulting in a partial dissociation of EcFicA_E28G_ from EcFicT.

### Structure determination of EcFicT/EcFicA complexes

**Fig 2:**
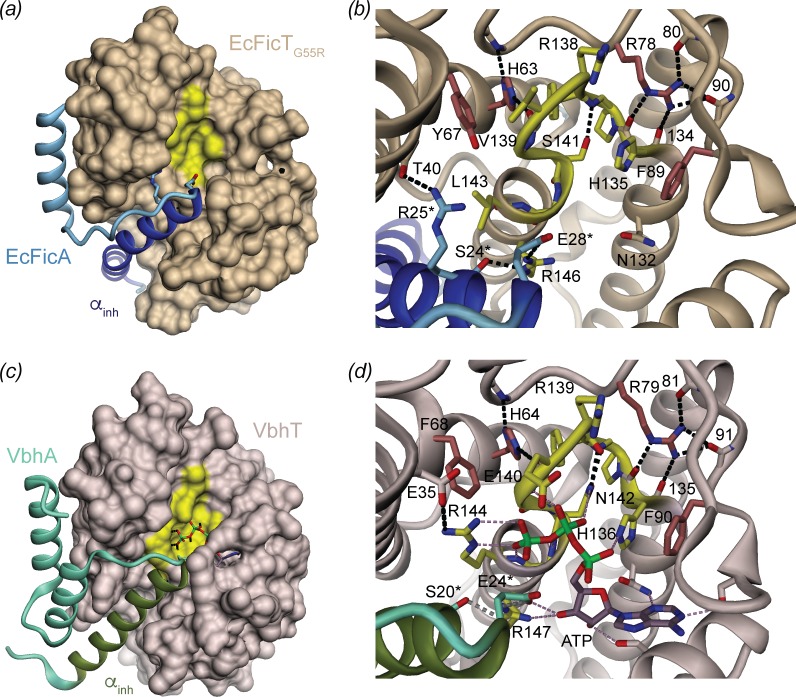
Structure comparison of EcFicT_G55R_/EcFicA with VbhT/VbhA. (*a*) Overall structure of the EcFicT_G55R_/EcFicA complex. (*b*) Close-up view of the EcFicT active site with the active-site loop highlighted in yellow. Residues S24, R25 and E28 of EcFicA form a lock via three H-bonds with residues R146 and T40 of EcFicT. (*c*) Overall structure of the VbhT/VbhA complex (PDB: 3ZC7 [13]). The ATP substrate is shown in full. (*d*) Close-up view of the active site of VbhTA. In (*a*) and (*c*), the Fic proteins are shown as solvent accessible surface representation and the antitoxins in cartoon representation with the α_inh_ highlighted by a stronger hue. In (*b*) and (*d*), the active sites are shown in detail with the signature motif in yellow. H-bonds are shown as dashed lines.

Crystallization trials of the wild-type EcFicTA complex did not yield any detectable crystal. However, the EcFicT_G55R_ mutant in complex with EcFicA (EcFicT_G55R_A) yielded crystals that diffracted to 2.0 Å. Crystals belonged to the Laue group 6/m with unit cell parameters a = 104.69 Å, b = 104.69 Å and c = 110.52 Å. Systematic absences along the c*-axis (0,0,*l*) indicated space groups P6_1_ or its enantiomorph P6_5_. Unit cell parameters and space group suggested two 1:1 complexes per asymmetric unit (2.60 Å^3^/Da). The structure of EcFicT_G55R_A was determined at 2.0 Å resolution (Table 1) by molecular replacement using the structure of VbhT (33% sequence identity to EcFicT) from the VbhTA complex (PDB: 3ZC7) as search model (Fig 2A). All possible space groups were tested for molecular replacement using PHASER [25]. The best solution (TFZ = 11.6 and LLG = 206) was obtained in space group P6_5_ yielding two EcFicT molecules in the asymmetric unit. Initial refinement (R_work_ = 43.6%, R_free_ = 47.0%) showed additional α-helical structure (positive Fo-Fc map) in the groove of the Fic protein next to the active site, where usually the α_inh_ helix is found [9,13]. After automatic model building of this part followed by several cycles of iterative model building and refinement, an almost complete full model of the EcFicT_G55R_A complex was obtained with R_work_ and R_free_ values of 17.7% and 20.4%, respectively. Final refinement statistics and completeness of the model are given in Table 2.

For EcFicT in complex with the antitoxin mutant EcFicA_E28G_, crystals of the monoclinic space group C2 were obtained that diffracted to 2.4 Å, containing three toxin-antitoxin complexes per asymmetric unit. The structure of EcFicTA_E28G_ was solved by molecular replacement using the previously determined structure of EcFicT_G55R_A (PDB code: 5JFF) as search model. Data collection statistics, refinement statistics and completeness of the model are given in Tables 1 and 2, respectively. For both structures determined in this study, the geometry of the final model was assessed using MolProbity [30] showing >99% of the residues in the core and allowed regions of the Ramachandran plot.

### Structure analysis of the EcFicT/EcFicA complexes

Despite distinct crystal packings, the structures of EcFicT_G55R_A and EcFicTA_E28G_ are virtually identical (root mean square deviation (rmsd) of 0.59 Å for 232 Cα-atoms). In particular, the interactions formed between the Fic protein and the antitoxin are the same, giving confidence that they are physiological and also occur in solution. Biological relevance of this interface was also corroborated by EPPIC (Evolutionary Protein-Protein Interface Classifier [31]) analysis. No other interfaces of potential biological significance were identified

EcFicT is highly similar to other Fic domain structures. In particular, EcFicT_G55R_ superimposes very well with the structure of VbhT from *B*. *schoenbuchensis* and BepA from *Bartonella henselae*, with an rmsd of 1.36 Å and 1.65 Å for 187 and 189 Cα-atoms, respectively. Noteworthy, the pairwise sequence identity of EcFicT with both VbhT and BepA is rather low (33% and 32%, respectively). EcFicT forms a seven α-helices bundle with the Fic signature motif located between α4 and α5. The flap is located between helices α2 and α3 and forms a beta-hairpin (S1A Fig) that has been previously shown [32] or predicted to be involved in target docking [13,33,34]. The Fic core, as defined by pfam (pfam 02661 [35]), includes residues 54–157 and comprises helices α2 to α5 (S1A Fig).

The EcFicA antitoxin tightly embraces EcFicT forming mainly hydrophobic interactions, as observed also in the VbhA/VbhT complex (Fig 2). Yet, EcFicA adopts a unique conformation, forming two parallel α-helices linked by a 10-residues loop (Fig 2A) compared to VbhA that forms three anti-parallel α-helices (Fig 2C). The second helix of VbhA is degenerated to a loop in EcFicA. Sequence alignment reveals an eight amino acids deletion in EcFicA compared to VbhA (S2 Fig). Furthermore, the first helix of EcFicA (α1) that is homologous to the α_inh_ helix is kinked and two amino acids longer than the corresponding helix of VbhA. The kink occurs at the same position as in PhD, the antitoxin of the Doc toxin from Bacteriophage P1, although the angle and direction are different (S1B Fig).

The SRRLEG segment at the C-terminal end of the first helix of EcFicA (Fig 2B) conforms to the canonical [S/T]xxxE[G/N] inhibition motif and protrudes into the EcFicT active site as in the VbhTA complex. S24 and E28 of EcFicA form, respectively, H-bond and salt-bridge interactions with R146 (R_2_) of the canonical Fic signature motif (compare Fig 2B with Fig 2D). Additionally, R25 of EcFicA interacts with T40 of EcFicT (Fig 2B). An interface of 1641 Å^2^ is formed between EcFicT and EcFicA, corresponding to a gain in free energy (Δ^i^G) of -19.6 kcal/mol upon formation of the interface as calculated by the PISA web server [36]. This interface is mainly hydrophobic: 13 residues of EcFicA (out of 33) are hydrophobic and contribute to a buried surface area of 727 Å^2^ and 17 residues of EcFicT (and out 44) are hydrophobic and contribute to a buried surface area of 710 A^2^. This large and hydrophobic interface is consistent with the stable complex that has been observed in solution (Fig 1).

### Phylogenetic analysis of EcFicT homologs

In order to obtain insight into the functional evolution of EcFicT, we generated a multiple sequence alignment comprising 702 sequences of EcFicT homologs down to 50% sequence identity, which was reduced to 102 sequences by manually removing redundant sequences (S3 Fig). Interestingly, these close EcFicT homologs all belong to *Enterobacteriaceae*, indicating that vertical transmission and not horizontal gene transfer played a major role in their evolutionary history. This hypothesis is strongly supported by the finding of largely syntenic loci for all close enterobacterial homologs of EcFicT, but no conserved genetic associations for other, distantly related, FicTA modules (S4 Fig). The inferred vertical transmission of the genes coding for EcFicT-like proteins contrasts with the situation found for type II toxin-antitoxin modules that, during evolution, are typically transmitted via horizontal gene transfer [37]. The same has been recently suggested for FIC domain proteins in general [38].

**Fig 3:**
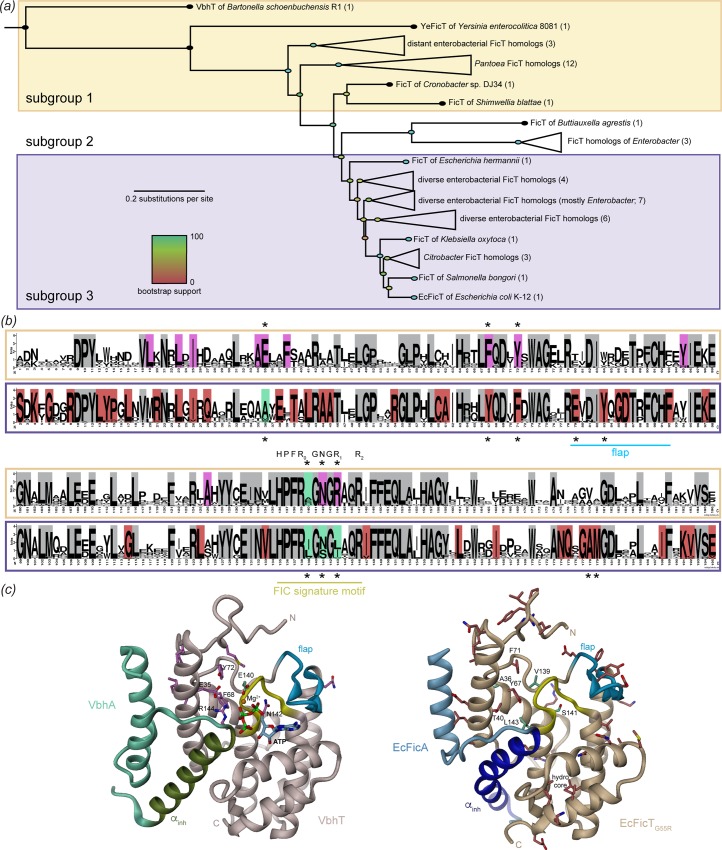
Phylogenetic and SDP analysis of EcFicT-like sequences. (*a*) Comprehensive phylogeny of representative sequences of the EcFicT-like subfamily of class I Fic proteins. The maximum likelihood phylogeny was rooted using the FIC domain of VbhT, a distantly related FicT protein of *B*. *schoenbuchensis*, as an outgroup and included the FIC domain of YeFicT of *Yersinia enterocolitica* str. 8081 FicT (YeFicT) for comparison [15]. Bootstrap support for individual branches is indicated by color code on the nodes. Yellow background coloring indicates the presence of a canonical FIC domain active site motif (subgroup 1), and the tree topology reveals that this is the ancestral state among the enterobacterial FicT homologs. The other sequences display different degrees of degeneration of the active site sequence (subgroups 2 and 3; see also S3 Fig). Among these, a group of FicT proteins of *Enterobacter* are hallmarked by a distinctive sequence variant at their N-terminus (in subgroup 2; no background coloring, see also S3 Fig). Purple background coloring refers to close homologs of EcFicT (subgroup 3) (*b*) Aligned weblogos of distant (subgroup 1, yellow) and close (subgroup 3, purple) EcFicT homologs, shown in the top and bottow row, respectively. Residues with a larger than 75% overall conservation are shown in grey and represents residues that do not confer subgroup specificity. Residues conserved by more than 75% but only within one of the subgroups are colored in magenta (top row) or red (bottom row) and indicate SDPs. Additionally, variable subgroup 3 residues homologous to positions important for the AMP-transferase activity of subgroup 1 members are highlighted in green. Residues discussed in the manuscript are indicated by stars. The flap and active site are indicated. (*c*) Specificity determining positions (SDPs) are shown in stick representation on the crystal structure of VbhTA (left, PDB: 3ZC7) and EcFicT_G55R_A (right). Note that ATP in competent conformation has been obtained by superimposition with the structure of VbhTA_E24G_ (PDB: 3CZB).

The phylogenetic relation between the sequences of the multiple sequence alignment as derived from a maximum-likelihood analysis is shown in S3 Fig, and a reduced version of the tree (after filtering out redundant sequences with > 90% identity) is given in Fig 3A. The phylogeny was rooted using VbhT, a very distantly related FicT toxin of *B*. *schoenbuchensis*, and additionally contains the distinct, yet enterobacterial YeFicT toxin for comparison [15]. These two proteins and ancestral branches of the tree (subgroup 1, yellow background in Fig 3A and S3 Fig) form a group of rather distant EcFicT homologs (sequence identity between 50% and 60%). Most group members harbor the asparagine and both arginines of the canonical HxFx[D/E]GNGR_1_xxR_2_ adenylylation-competent motif, and the AMPylation activity of VbhT has been demonstrated [15]. Subgroup 2 (shown in the middle of the tree) is characterized by a specific pattern at the N-terminus. While all the analyzed sequences are of very similar length, a five amino-acids deletion appears at the N-terminus of subgroup 2 sequences. Conspicuously, this group and the EcFicT-like homologs (subgroup 3; displayed at the bottom of the tree with purple background in Fig 3A and S3 Fig) have signature motifs that differ from the ancestral state in subgroup 1. Compared to the canonical sequence, a small to medium-sized hydrophobic residue invariably substitutes arginine R_1_ that in AMP transferases is heavily involved in ATP binding [9,13] suggesting that these proteins are not adenylylation-competent and may have evolved a new biochemical function.

### Specificity determining positions that distinguish EcFicT from canonical FIC AMP transferases

To validate the subgrouping derived from the phylogenetic analysis and to systematically identify subgroup defining positions (SDPs), we submitted the multiple sequence alignment to the SPEER server (specificity prediction using amino acid’s properties, entropies and evolution rate [18]). Four subgroups (I–IV) were identified by SPEER as indicated in S3 Fig that matched almost perfectly the grouping derived from the phylogenetic analysis, though with subgroup 1 being divided in two parts (SPEER groups II and IV).

To reveal the SDPs, we computed the sequence logos for the subgroups of canonical AMP transferases (subgroup 1) and of EcFicT-like proteins (subgroup 3), which are displayed in Fig 3B in the top and bottom row, respectively. While about 40% of the residues are almost strictly conserved amongst the two subgroups (marked in grey on Fig 3B), there are several residues that are conserved only in one or the other subgroup (marked with magenta or red background, respectively). These SDPs are found throughout the entire sequence and their number is considerably larger in the EcFicT-like than in the canonical subgroup (12 magenta *vs*. 43 red positions). Possibly, this reflects additional evolutionary constraints on the structure and function of the EcFicT-like proteins. While many of the SDPs correspond to buried residues (see also Fig 3C) that, thus, would have maintained their specificity for structural reasons, there are also several positions that are exposed and probably related to function. Latter positions map to (i) the canonical ligand-binding pocket, (ii) the flap that is expected to interact with a putative target, and (iii) the N-terminus (Fig 3C). Some additional positions were manually identified (shown in green). These positions are slightly variable in each subgroup and, therefore, not picked up automatically using a sequence identity threshold.

Consistent with our preliminary assignment as canonical AMP transferases, subgroup 1 exhibits an almost canonical HPFR_0_(D/E)GNGR_1_xxR_2_ signature motif. The R_1_ position is clearly a SDP for that group and contrasts strongly with the small to medium-sized hydrophobic residue in EcFicT-like proteins (subgroup 3). The conserved central asparagine residue is found mutated to a serine in about half of the EcFicT-like sequences. The full phylogeny of subgroup 1 (S3 Fig) reveals that this asparagine to serine mutation has occurred several times independently. An asparagine or a serine can most likely adopt this position without altering the H-bond interaction with the main-chain amide of the strictly conserved structural phenylalanine of the signature motif (F137 of EcFicT). Unexpectedly, the D/E residue of the canonical motif (E140 in VbhT) that is involved in Mg^2+^ coordination in known structures (Fig 3C, left) is not well conserved in subgroup 1. It would be interesting to further functionally characterize this group with respect to magnesium requirement. In subgroup 3, the equivalent position is almost invariably a leucine or isoleucine.

There are several additional SDPs close to the site that binds ATP in VbhT (subgroup 1, Fig 3C). In VbhT, the aforementioned R_1_ (R144) is close to F68; in EcFicT, the corresponding residues are L143 and Y67 (Fig 3C). In VbhT, a tyrosine at position 68 would clash with arginine R_1_. It is noteworthy, that Y67 of EcFicT is interacting indirectly with R25 of the antitoxin via a water molecule. Removal of the antitoxin may open up a binding site for a yet unidentified substrate potentially involving this tyrosine.

**Fig 4:**
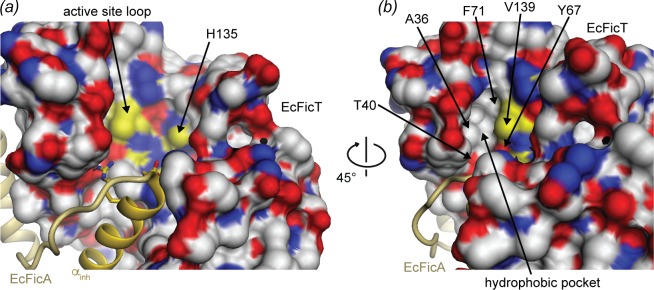
Putative hydrophobic substrate-binding pocket of EcFicT. (*a*) EcFicTA structure in a similar view and representation as in Fig 2A, but with positively charged atoms colored in blue and negatively charged atoms colored in red. Hydrophobic atoms are shown in white, except atoms of the active site loop that are highlighted in yellow. (*b*) A rotation of 45°C around a vertical axis reveals an additional hydrophobic pocket.

Strikingly, a highly conserved glutamate of subgroup 1 (E35 of VbhT) is mutated to alanine (A36 of EcFicT) in most subgroup 3 sequences. The glutamate interacts with R_1_ and thus, the glutamate to alanine change may represent co-variation with the R_1_ to hydrophobic amino acid mutation (Figs 3C and 4). Finally, a strict tyrosine to phenylalanine change is apparent in position 72 of VbhT (71 in EcFicT), bordering the signature loop. In summary, next to the signature loop, EcFicT-like proteins exhibit an extended groove lined by hydrophobic residues (A36, F71, V139) and bordered by the polar residues Y67 and T40 with exposed hydroxyl groups (Fig 4). All 5 positions are SDPs, *i*.*e*. specifically conserved is subgroup 3. It is well conceivable that EcFicT-like proteins have evolved this binding pocket to accommodate a substrate distinct from ATP used by canonical AMP transferases (S5 Fig).

**Fig 5:**
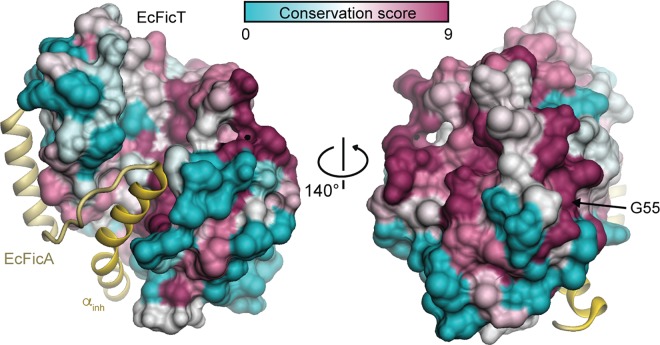
Sequence conservation of EcFicT. Surface representation of EcFicT colored by conservation with a cyan-white-purple gradient for variable to conserved residues. EcFicA is shown in yellow with the α_inh_ helix in gold. The strictly conserved residue G55 is indicated.

Also at the flap and close to it, several residues are well-conserved in the EcFicT-like subgroup. In particular, E79 and Y83 are interesting, since they are exposed and may provide specificity for the interaction with a yet unidentified target protein. The strictly conserved alanine and methionine at positions 178 and 179, respectively, are located just below the flap and may thus be important for proper positioning of the flap for target recognition and registration of the modifiable segment. The overall analysis of the conservation of amino acids at the surface of EcFicT-like Fic proteins reveals two well-conserved patches: the first one located below the flap (residues 123, 126, 130, 133, 175 and 178–180) and a second one on the “back” of the Fic protein (residues 92, 96, 100, 103, 107), see Weblogo on Fig 3B and ConSurf representation on Fig 5. These two patches may be involved in target recognition of a subgroup specific target that remains to be discovered.

### Putative regulation of FicT proteins by tyrosine auto-modification

**Fig 6:**
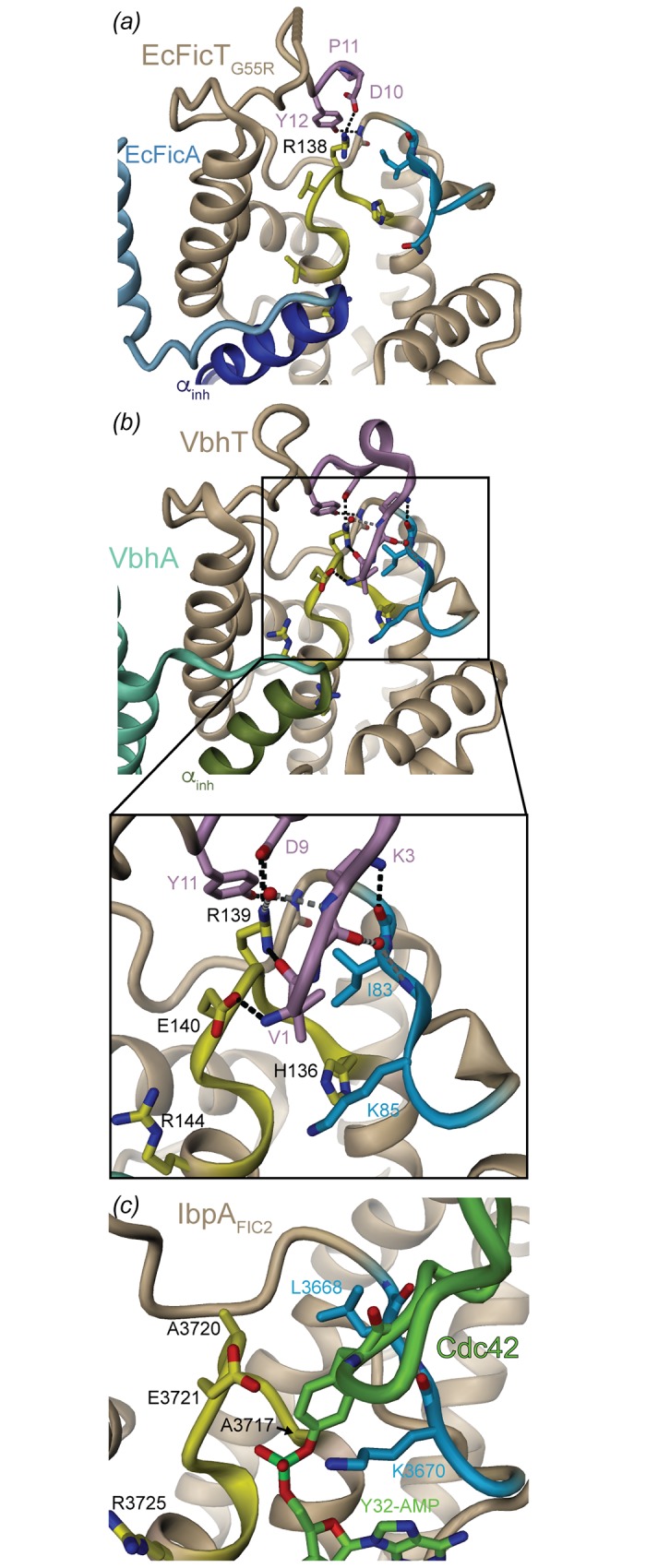
N-terminal lid-lock of class I Fic proteins. (*a*) EcFicT_G55R_A structure depicted as cartoon representation with EcFicT_G55R_A colored in beige (active site loop in yellow, flap in light blue). The DPY motif (residues 10–12) is colored in pink. EcFicA is colored in blue. (*b*) The apo structure of VbhTA (PDB: 3SHG [9]) is depicted in a similar view and representation as EcFicT_G55R_A in panel (*a*). VbhA is colored in green. The N-terminal VbhT segment is colored in pink. Note that a tartaric acid molecule is bound to the active site of VbhT (not shown). In (*a*) and (*b*), H-bonds between the N-terminal segment and the flap or active site residues are shown as dashed lines. (*c*) For comparison, interaction of the modifiable segment of Cdc42 to the flap of IbpA_Fic2_ in the IbpA_Fic2_/Cdc42 complex structure (PDB: 4ITR [32]).

The sequence logos of Fig 3B reveal a DPY motif that is strictly conserved in subgroups 1 and 3. In the crystal structure of EcFicTA, the first eight amino acids of EcFicT are unresolved, but the structure of the DPY motif (residue numbers 10–12) is resolved. Fig 6A shows that the aspartate (D10) and tyrosine (Y12) side-chains interact with the side-chain of arginine R138 (4^th^ residue of the Fic signature motif, referred to as R_0_) and the main-chain amide of residue 79 (preceding the flap), respectively. The same organization can be seen in the apo VbhTA crystal structure [9]. This interaction may be crucial for the observed fixation of the VbhT N-terminus to the flap (Fig 6B), which is the docking site for the modifiable segment of the target as *e*.*g*. observed in IbpA_FIC2_/Cdc42 (Fig 6C) [32] and inferred from other Fic structures that show a chain-terminus from an adjacent Fic molecules in the crystal bound to this site [13].

The interaction of the N-terminus with the target dock, which had been over-looked so far, may constitute a physiological auto-inhibition mechanism (N-terminal lock), since, obviously, it would impede productive target binding.

In analogy to the relief of auto-inhibition exerted by the auto-modification of the α_inh_ in NmFic and probably most of class III Fic proteins [22], it is tempting to speculate, that also the conserved tyrosine of the DPY motif can get auto-modified to relieve auto-inhibition. It is striking that R_0_ of the Fic motif is highly variable in adenylylating Fic enzymes in general, but strictly conserved in subgroups 1 and 3 of Enterobacterial class I Fic proteins (see [15] and also S3 Fig). Noteworthy, the DPY motif is absent in subgroup 2 sequences (S3 Fig), which correlates perfectly with the residue homologous to R_0_ being a non-arginine. Thus, the conservation of R_0_ in subgroups 1 and 3 may indeed indicate its functional role in the N-terminal lock stabilization.

### Effect of the EcFicT_G55R_ mutation

**Fig 7:**
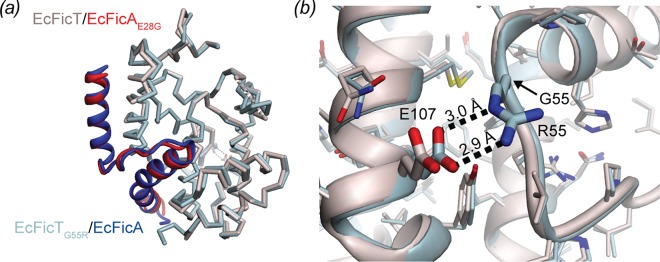
Structural comparison of EcFicTA_E28G_ and EcFicT_G55R_A. (*a*) Superimposition of the two structures determined in this study reveals a well-conserved Cα-trace with an rmsd of 0.55 Å for 229 atom pairs of the EcFicTA complexes. (*b*) Close-up view of the salt-bridge formed between R55 and E107 in the EcFicT_G55R_A complex. Note that the two crystal structures were obtained from two different crystal forms (space groups P6_5_ and C2 for EcFicT_G55R_A and EcFicTA_E28G_, respectively; see Table 1 for details).

Our crystal structures show that the G55R mutation encoded by the *fic-1* allele, which results in the cell filamentation phenotype [3], does not affect the structure of EcFicT (see above and Fig 7A). The Ramachandran angles of R55 (Φ, Ψ = -120.7, -160.4) are virtually the same as those of the G55 wild-type residue (Φ, Ψ = -133.6, -155.8 as measured in the EcFicTA_E28G_ structure). The arginine 55 side-chain forms a salt-bridge with glutamate 107 of helix α3 (Fig 7B), which is conserved in EcFicT-like homologs (Fig 3B). One could speculate that the glycine to arginine mutation may reduce the main-chain flexibility. However, despite the additional salt-bridge, the G55R variant is not more stable than the wild-type protein, as has been shown above by DSF (Fig 1B).

### Effect of the EcFicA_E28G_ mutation

EcFicTA_E28G_ crystallized in the space group C2 with three complexes per asymmetric unit. The structure of this mutant is virtually identical to that of EcFicT_G55R_A, except for disorder of residues 83–86 at the tip of the flap. In EcFicT_G55R_A, the flap is stabilized by crystal contacts and is fully ordered. The lack of electron density of the flap of the E28G mutant has already been observed in several other FIC domain protein structures (*e*.*g*. Bep1 from *Bartonella clarridgeiae*, PBD: 4NPS or NmFic_E102R_ from *Neisseria meningitidis*, PDB: 5CGL). We speculate that, in solution, this region gets stabilized only upon substrate and/or target binding and that it is likely to be disordered for EcFicT_G55R_A too.

In canonical AMP-transferases, the conserved glutamate of α_inh_ is engaged in a salt-bridge with arginine R_2_ of the Fic motif, thereby preventing competent ATP binding. Also in EcFicT_G55R_A, such an intermolecular salt-bridge is observed (E28–R146). Though the side-chain truncation in EcFicA_E28G_ increases the size of the putative substrate binding site, it may not be sufficient to allow productive substrate binding and relieve the inhibition of EcFicT. Because of the hydrophobic pocket on the left-hand side of the active site that may accommodate a different substrate, de-binding of EcFicA or more drastic mutations may be required to allow ligand binding.

## Discussion

The Fic protein from *E*. *coli* (now named EcFicT) was identified more than 30 years ago [1] and was shown to be responsible for the filamentation induced by cAMP (fic) phenotype when carrying a G55R amino acid exchange (encoded by the *fic-1* allele), but its molecular and biological function remained elusive.

Since then, a number of Fic proteins from other organisms have been shown to catalyze adenylylation of target proteins, resulting in modulation of their functions, *e*.*g*. by inhibiting Rho-GTPase pathways or bacterial DNA topoisomerases [6,7,15,39]. The vast majority of adenylylation-competent Fic proteins are expressed in an inhibited form [9]. The location of the inhibition motif lead to the classification of Fic proteins in class I, II and III depending on this motif being contributed by an antitoxin, or found at the N-terminal or C-terminal end of the Fic protein, respectively. According to this classification, EcFicT belongs to class I with the inhibitory helix provided by EcFicA, a small protein coded immediately upstream of the *ecficT* gene. The synteny of the corresponding genetic loci reveals that the genes were primarily transmitted vertically within the *Enterobacteriaceae*, suggesting a specific physiological function. This is in contrast to typical type II TA modules that show considerable mobility via horizontal gene transfer, likely related to their biological functions in the context of mobile genetic elements [40].

The crystal structure of the tight EcFicTA complex reported in this study shows that the EcFicT protein adopts the canonical fold with a somewhat degenerated signature motif and that it is embraced by the cognate EcFicA antitoxin very similarly as in the AMP-transferase VbhTA complex [9]. The inhibitory glutamate of EcFicA forms a salt-bridge with the last arginine (R_2_) of the Fic motif of EcFicT, as in VbhTA (Fig 2B and 2D). Despite the close similarity, the structure suggests that EcFicT most likely does not bind ATP, consistent with the lack of any evidence for auto-adenylylation [15]. In this respect, the presence of a leucine residue instead of the canonical arginine R_1_ appears most relevant. R_1_ interacts with the β-phosphate of the ATP substrate in the inhibition relieved form of VbhT [13] and with the α-phosphate of CDP-choline in AnkX [41] (S5 and S6 Figs). Still, in EcFicT, binding of a diphosphate moiety to the anion-binding nest at the N-terminus of the helix appears still sterically feasible due to the conservation of the second glycine of the signature motif (Fig 3B). Another salient difference seen in EcFicT is the presence of a hydrophobic residue at the position following R_0_ (Fig 3B), which in canonical FIC AMP-transferases is taken by a charged residue to form a direct (lysine, PDB: 4WGJ, SSGCID) or indirect (glutamate via a Mg^2+^ ion) interaction with substrate phosphate(s). The adenine-binding pocket as observed in VbhT appears present in EcFicT and the asparagine preceding the Fic signature motif is strictly conserved in EcFicT homologs (subgroup 3, Fig 3B). In the EcFicT_G55R_A structure, the base-binding pocket is nevertheless partly restricted by the conformation that adopts the glutamine 84 of EcFicT located at the tip of the flap. The flap is a rather flexible part of the protein and likely only stabilizes upon target binding or artificially by crystal contacts in x-ray structures. An alternative rotamer and/or a slight different conformation of the flap would result in an unrestricted base-binding pocket.

The comparative analysis of EcFicT-like and Fic AMP-transferase sequences not only showed that the two aforementioned charged residues of AMP-transferases are replaced by hydrophobic residues in EcFicT-like proteins, but that there are about a dozen subgroup defining positions (SDPs) (Fig 3). Since many SDPs of the EcFicT subgroup are localized close to the signature motif, it appears likely that they are involved in binding of a yet unidentified substrate. If in addition R_2_ were also involved in substrate binding, its interaction with the inhibitory antitoxin glutamate may again, as in *e*.*g*. VbhTA toxin-antitoxin complex, compromise substrate binding and, thus, inhibit the putative catalytic activity. Additional SDPs of EcFicT-like proteins cluster at the exposed face of the flap and in the N-terminal segment (Fig 3C, right). These positions may well be conserved for subgroup-specific recognition of a putative target protein.

Phylogenetic analysis showed that EcFicT has evolved out of canonical AMP transferases in the Fic protein family (Fig 3A and S3 Fig). Considering the well-defined and conserved structural features of EcFicT-like proteins, we suggest that during evolution the Fic structure has been reshaped to accommodate a yet unidentified substrate, and to recognize a new range of targets. Interestingly, the histidine, crucial for deprotonation of the incoming target side-chain hydroxyl group, remains conserved in EcFicT-like proteins.

Class I Fic proteins in general show a conserved DPY segment that seems to tether the N-terminal segment to the body of the FIC domain as observed in EcFicT, but also in the VbhT structure (Fig 6A and 6B). Interestingly and underappreciated in our previous work [9], the very N-terminus of VbhT (which is unresolved in the EcFicT structure) is laterally bound to the flap, thereby augmenting the small β-sheet, in a similar manner as observed for target peptide binding (Fig 6C) [32]. This finding suggests that class I Fic protein may have, apart from the inhibitory glutamate preventing competent substrate binding, another auto-inhibitory element competing with target binding. In the light of recent reports that auto-adenylylation can relieve auto-inhibition in Fic proteins [22,42], we note that the conserved tyrosine of the N-terminal DPY motif (Y12 of EcFicT) is buried (Fig 6A) and that, therefore, its modification with a bulky group may interfere with binding of the N-terminal segment to the target dock and therefore relieve inhibition.

The “filamentation induced by cAMP” phenotype is hallmarked by the inhibition of cell division in an environment of elevated temperature and cAMP concentrations (43°C) and caused by the *fic-1* allele of *ecficT* [1]. The EcFicT_G55R_A crystal structure revealed that the site of mutation is neither close to the presumed active site nor the presumed target recognition site. The comparison of the thermal stability of EcFicTA and EcFicT_G55R_A revealed the same melting temperature for both complexes (Fig 2B). But, once dissociated from its cognate partner EcFicA, the melting temperature of EcFicT may be more severely decreased than the one of EcFicT_G55R_. Furthermore, it has been suggested in a recent review [43] that the G55R mutation may result in "long-range conformational changes that facilitate the dissociation of the corresponding antitoxin". However, we did not observe such effects as the EcFicTA_E28G_ and EcFicT_G55R_A structures are very similar. Also, the oligomeric state of the EcFicTA protein complex is not affected by the mutation (Fig 2A). Nonetheless, we cannot exclude that upon unbinding of the antitoxin, long-range structural changes may occur. Noteworthy, the Doc toxin (mutant H66Y) forms a sub-domain swapped dimer in absence of its cognate PhD antitoxin [44]. Our structural work does not provide an explanation or potential mechanism for the results obtained for the originally described FIC domain protein EcFicT [1]. Further experiments, such as ligand docking combined with biochemical analyses, may help revealing the role of enterobacterial FIC-domain proteins.

## Conclusion

The *E*.*coli* Fic protein EcFicT forms a tight complex with its cognate antitoxin EcFicA. EcFicT adopts the conserved Fic fold, but with a distinct active site signature motif and an additional hydrophobic binding pocket in the vicinity of the active site loop. EcFicA forms two parallel α-helices that tightly embrace EcFicT. The structural information provided in this manuscript may serve as a basis to identify new potential substrates of Fic proteins with EcFicT-like active site motif, if combined with the appropriate bioinformatical ligand docking experiments. Still, further *in silico*, *in vitro* and/or *in vivo* analyses are required to understand the activity of the first described FIC domain protein.

The combination of cognate FicA antitoxins and the N-terminal lock may form a double-lock mechanism of regulation for class I Fic proteins. This may be analogous to the double-lock mechanism of class III Fic proteins via oligomerization and auto-adenylylation that we have recently described [22]. Given that all structural features required for the two mechanisms of catalysis regulation are fully conserved in the EcFicT-like subfamily, we find it likely that these proteins are indeed enzymes whose catalytic activity merely remains to be uncovered.

Taken together, we propose that the EcFicT-like subfamily of class I Fic proteins has adapted the conserved FIC domain fold for a new (possibly catalytic) molecular activity in the context of a biological function outside the framework of classical FicTA toxin-antitoxin modules that disrupt DNA topology [15].

## Supporting Information

S1 Fig Comparison of the antitoxin-binding mode in the EcFicTA and Doc/PhD complexes. (*a*) Cartoon representation of the EcFicT_G55R_A complex. EcFicT_G55R_ is colored in beige with the active site loop highlighted in yellow and EcFicA is colored in light blue with the α_inh_ helix highlighted in dark blue as in Fig 2A. (*b*) Cartoon representation of the Doc/PhD complex in the same orientation as the EcFicT_G55R_A complex shown in panel *a*. Doc is colored in light brown with the active site loop highlighted in yellow and PhD is colored in sea green. Doc is a homolog of EcFicT and both proteins belong to the Fido protein family [45]. Note that the kink in the helix of the antitoxin occurs at the same position in both rather distant complexes.
(TIF)

S2 Fig Protein sequence alignment of VbhA and EcFicA. The inhibition motif is depicted by a yellow rectangle and the position of the α-helices as observed in the crystal structures are depicted as blue cylinders. The residue numbers correspond to the amino acids numbers in each protein sequence. Red lines indicate structurally equivalent residues.
(TIF)

S3 Fig Full alignment of Enterobacterial EcFicT homologs. The phylogeny of Fig 3A is shown in an extended view (left) for comparison with a full-length alignment of the respective protein sequences (right). Though some inner branchings of the phylogeny are not well supported, the tree topology clearly shows that the mutation of asparagine to serine at the center of the active site loop occurred several times independently (red arrow). The four subgroups (I–IV) identified by the analysis of the multiple sequence alignment using the SPEER server are indicated on the left-hand side. Furthermore, the remarkable conservation of a tyrosine at the N-terminus of FicT proteins is clearly apparent (black arrow). The alignment also shows the distinctive N-terminal region of a FicT subgroup in *Enterobacter* (no background coloring) that does not contain the aforementioned tyrosine.
(TIF)

S4 Fig Analysis of the genomic context of *ficTA* loci. The illustration shows the loci encoding diverse FicTA modules. A small gene (colored in blue) is located directly upstream of the fic gene (colored in red), corresponding to EcFicA. Note that a *ecFicA* homolog is encoded upstream all *ecFicT*, except in the sequence of *Escherichia albertii TW07627* as a result of a single point mutation that introduced a stop codon (indicated by a blue star). Furthermore, the position of *ficTA* loci between *ppiA/B* and *pabA* is conserved for all close enterobacterial homologs of EcFicA (top) while no relevant patterns of synteny can be observed for the other FicTA loci. Enterobacterial FicT homologs with syntenic loci are displayed above the dotted line.
(TIF)

S5 Fig Surface representation of FIC domain proteins bound to their respective ligands. (*a*) VbhT/VbhA_E24G_ in complex with ATP using the same color code as in Fig 2C with the active site loop highlighted in yellow (PDB: 3CZB [13]). (*b*) AnkX in complex with CDP-choline (PBD: 4BET [41]). (*c*) Apo EcFicTA_E28G_. Note that the active site pocket is extended on the side of the base-binding site shown in panel *b*.
(TIF)

S6 Fig Comparison of the active site of EcFicT and Fic proteins with various known substrates. The loop corresponding to the Fic signature motif is highlighted in yellow. Residues involved in diphosphate binding (*b-d*) or their structural homologs (*a*) are shown in full. (*a*) EcFicT_G55R_ colored in beige with EcFicA colored in blue. (*b*) VbhT (beige) and VbhA_E24G_ (pink) in complex with ATP (PDB: 3ZCB [13]). (*c*) AnkX in complex with CDP-choline (PDB: 4BET [41]). The flap is colored in light blue. (*d*) Apo Doc/PhD complex (PDB: 3KH2 [46]) with PhD colored in sea green as in S1B Fig.
(TIF)
