# Supplementary figures and images for: Crystal Structure of the Escherichia coli Fic Toxin-Like Protein in Complex with Its Cognate Antitoxin
Source: PLoS One. 2016 Sep 22;11(9):e0163654. doi: 10.1371/journal.pone.0163654 (PMC5033356; doi:10.1371/journal.pone.0163654)

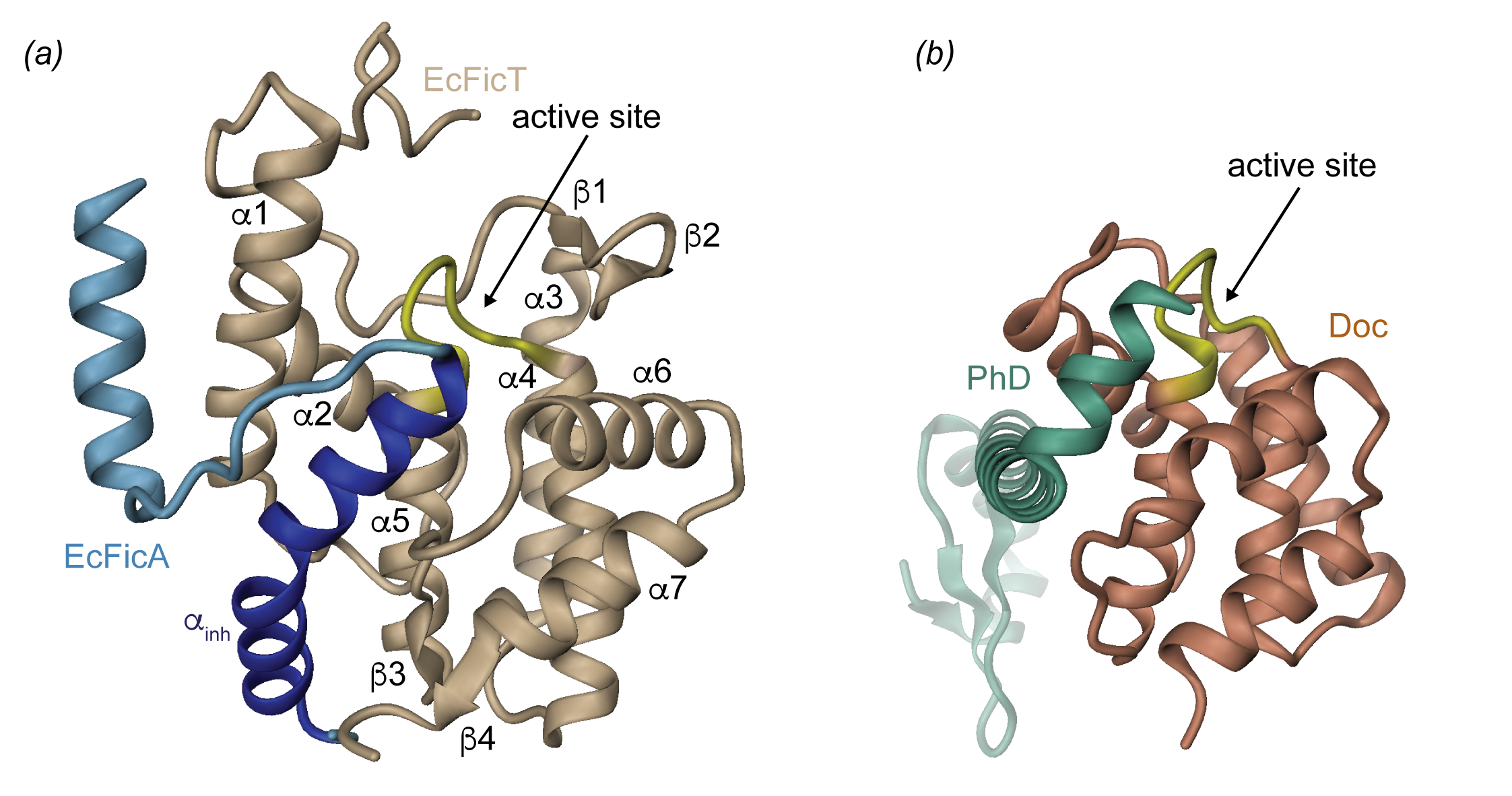

Supplement: S1 Fig — (a) Cartoon representation of the EcFicTG55RA complex. EcFicTG55R is colored in beige with the active site loop highlighted in yellow and EcFicA is colored in light blue with the αinh helix highlighted in dark blue as in Fig 2A. (b) Cartoon representation of the Doc/PhD complex in the same orientation as the EcFicTG55RA complex shown in panel a. Doc is colored in light brown with the active site loop highlighted in yellow and PhD is colored in sea green. Doc is a homolog of EcFicT and both proteins belong to the Fido protein family [45]. Note that the kink in the helix of the antitoxin occurs at the same position in both rather distant complexes. (TIF) [file pone.0163654.s001.tif]

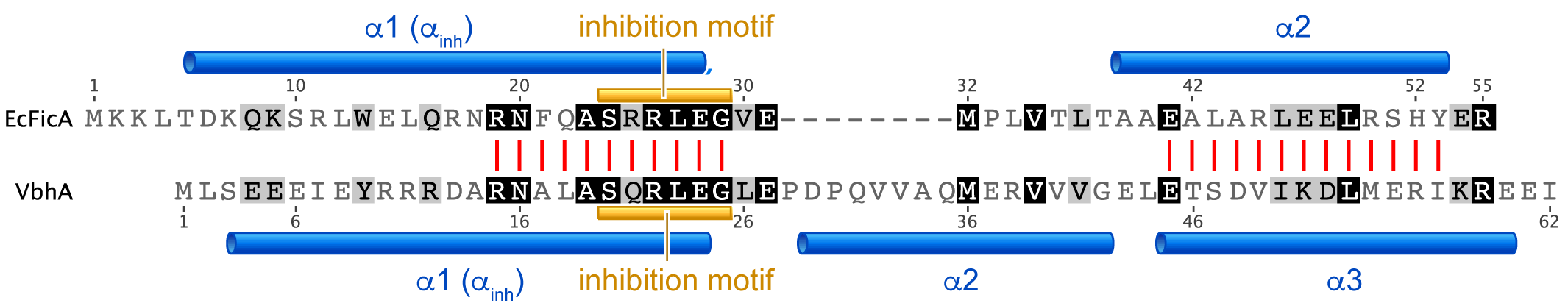

Supplement: S2 Fig — The inhibition motif is depicted by a yellow rectangle and the position of the α-helices as observed in the crystal structures are depicted as blue cylinders. The residue numbers correspond to the amino acids numbers in each protein sequence. Red lines indicate structurally equivalent residues. (TIF) [file pone.0163654.s002.tif]

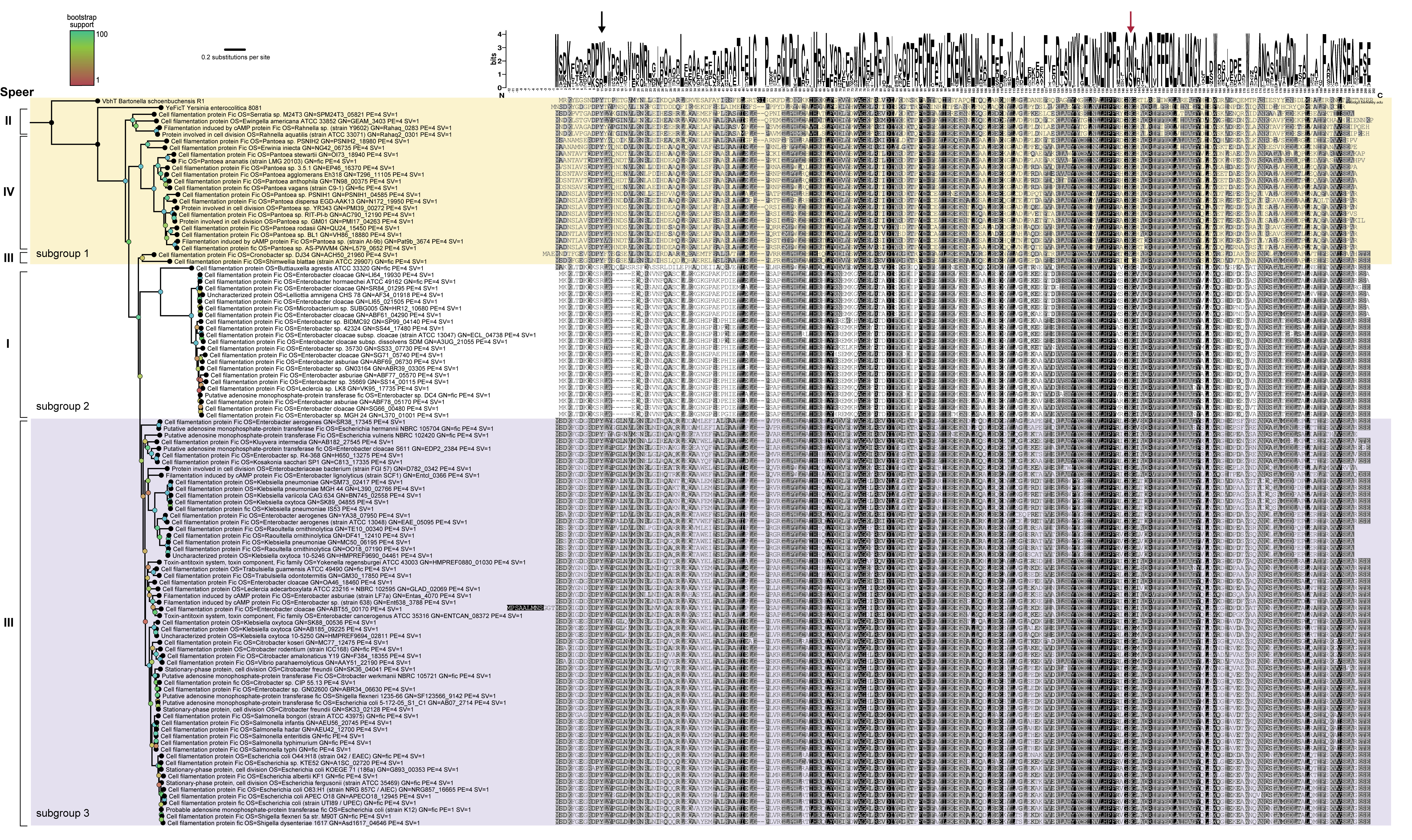

Supplement: S3 Fig — The phylogeny of Fig 3A is shown in an extended view (left) for comparison with a full-length alignment of the respective protein sequences (right). Though some inner branchings of the phylogeny are not well supported, the tree topology clearly shows that the mutation of asparagine to serine at the center of the active site loop occurred several times independently (red arrow). The four subgroups (I–IV) identified by the analysis of the multiple sequence alignment using the SPEER server are indicated on the left-hand side. Furthermore, the remarkable conservation of a tyrosine at the N-terminus of FicT proteins is clearly apparent (black arrow). The alignment also shows the distinctive N-terminal region of a FicT subgroup in Enterobacter (no background coloring) that does not contain the aforementioned tyrosine. (TIF) [file pone.0163654.s003.tif]

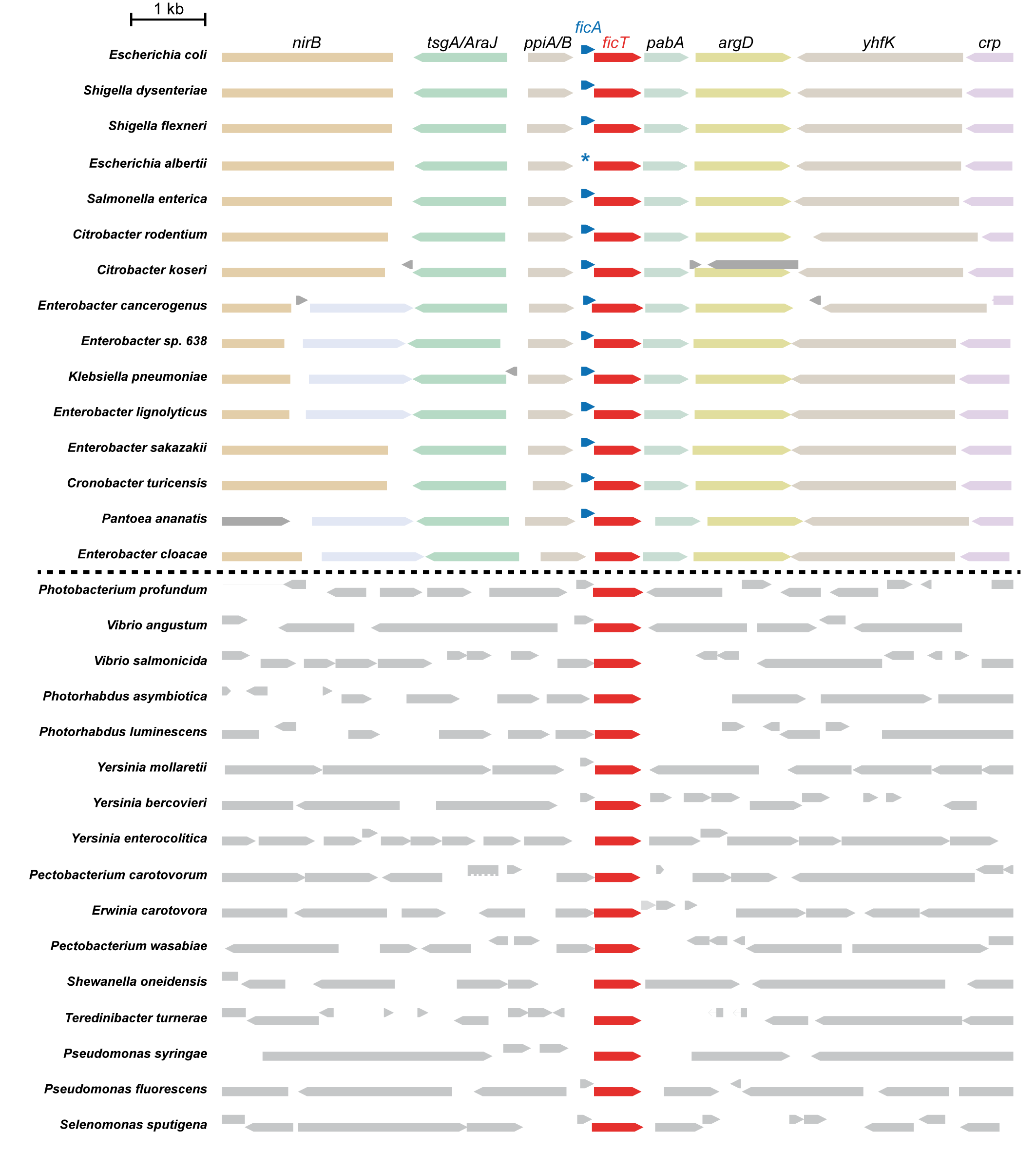

Supplement: S4 Fig — The illustration shows the loci encoding diverse FicTA modules. A small gene (colored in blue) is located directly upstream of the fic gene (colored in red), corresponding to EcFicA. Note that a ecFicA homolog is encoded upstream all ecFicT, except in the sequence of Escherichia albertii TW07627 as a result of a single point mutation that introduced a stop codon (indicated by a blue star). Furthermore, the position of ficTA loci between ppiA/B and pabA is conserved for all close enterobacterial homologs of EcFicA (top) while no relevant patterns of synteny can be observed for the other FicTA loci. Enterobacterial FicT homologs with syntenic loci are displayed above the dotted line. (TIF) [file pone.0163654.s004.tif]

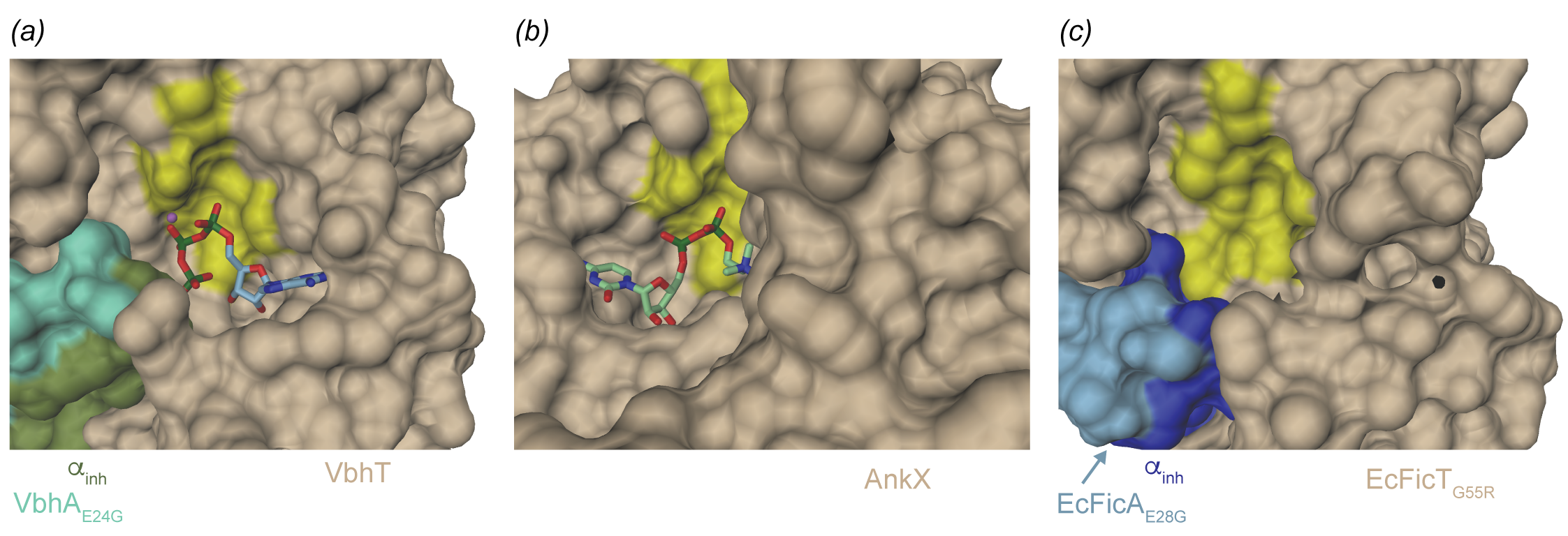

Supplement: S5 Fig — (a) VbhT/VbhAE24G in complex with ATP using the same color code as in Fig 2C with the active site loop highlighted in yellow (PDB: 3CZB [13]). (b) AnkX in complex with CDP-choline (PBD: 4BET [41]). (c) Apo EcFicTAE28G. Note that the active site pocket is extended on the side of the base-binding site shown in panel b. (TIF) [file pone.0163654.s005.tif]

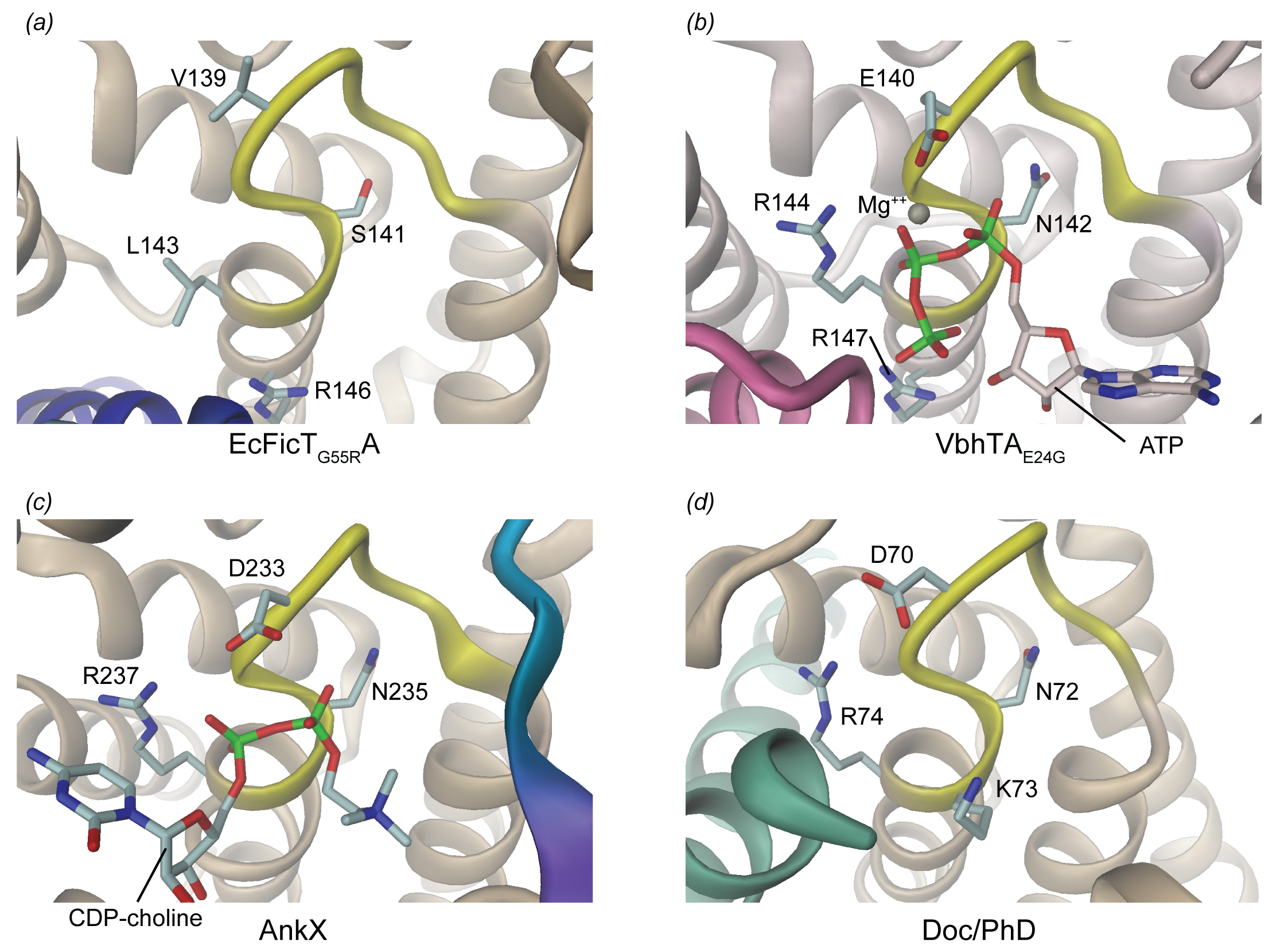

Supplement: S6 Fig — The loop corresponding to the Fic signature motif is highlighted in yellow. Residues involved in diphosphate binding (b-d) or their structural homologs (a) are shown in full. (a) EcFicTG55R colored in beige with EcFicA colored in blue. (b) VbhT (beige) and VbhAE24G (pink) in complex with ATP (PDB: 3ZCB [13]). (c) AnkX in complex with CDP-choline (PDB: 4BET [41]). The flap is colored in light blue. (d) Apo Doc/PhD complex (PDB: 3KH2 [46]) with PhD colored in sea green as in S1B Fig. (TIF) [file pone.0163654.s006.tif]
